# A comparative UPLC‐Q‐Orbitrap‐MS untargeted metabolomics investigation of different parts of *Clausena lansium* (Lour.) Skeels

**DOI:** 10.1002/fsn3.1841

**Published:** 2020-10-03

**Authors:** Ruiyi Fan, Cheng Peng, Xinxin Zhang, Diyang Qiu, Genlin Mao, Yusheng Lu, Jiwu Zeng

**Affiliations:** ^1^ Institute of Fruit Tree Research, Guangdong Academy of Agricultural Sciences Key Laboratory of South Subtropical Fruit Biology and Genetic Resource Utilization (MOA) Guangdong Province Key Laboratory of Tropical and Subtropical Fruit Tree Research Guangzhou China

**Keywords:** biomarkers, *Clausena lansium* (Lour.) Skeels, comparative analysis, different tissues, non‐targeted metabolomics, wampee

## Abstract

In this study, the non‐targeted large‐scale plant metabolomics (UPLC‐Q‐Orbitrap‐MS) was performed for the comparison of chemical profiling of the leaves, barks, flowers, peels, pulps, and seeds of *Clausena lansium* (Lour.) Skeels (called “wampee”). A total of 364 metabolites were identified, and 62 potential biomarkers were selected by the multivariate statistical analysis. Hierarchical cluster analysis suggested that the selected biomarkers were significant differential metabolites among various parts of wampee. Metabolic pathway analysis showed a significant enrichment of the “Flavone and flavonol synthesis” and “Isoquinoline alkaloid biosynthesis” pathway. This study provides important information for the isolation and identification of functional components from different tissues of wampee and the metabolic biosynthesis pathway elucidation in detail.

## Graphical abstract

The comparison of chemical profiling of the leaves, barks, flowers, peels, pulps, and seeds of wampee was investigated. This study provides important information for the isolation and identification of functional components from different tissues of wampee and the metabolic biosynthesis pathway elucidation in detail.

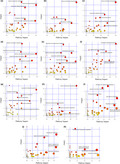

## 1 INTRODUCTION

*Clausena lansium* (Lour.) Skeels, also known as wampee, is one member of the Rutaceae originated from China and now widely cultivated in Guangdong, Guangxi, Hainan, Fujian, and Taiwan provinces (Fan et al., 2018). It has been reported that wampee is also planted in other countries such as the United States, Australia, India, and Sri Lanka (Chang, Lu, et al., 2018; Chang, Ye, et al., 2018). As an edible and medicinal fruit (Yang, Chen, & Huang, 1988), the bioactivities of the leaves, stem barks, seeds, and fruits of wampee have been investigated recently. For instance, the polyphenol extracts from the leaves of wampee were proven to have antidiabetic and lipid‐lowering effects on streptozotocin‐induced type 2 diabetic rats (Kong, Su, Guo, Zeng, & Bi, 2018). Chang, Lu, et al. (2018)) and Chang, Ye, et al. (2018) also investigated the polyphenolics profile and antioxidant activity of wampee leaves during the development process. Condensed tannins from the bark of wampee were reported to be capable of anti‐α‐glucosidase and antityrosinase (Chai et al., 2019). In recent years, a number of carbazole alkaloids and furanocoumarins which exhibited hepatoprotective and antiproliferative activities were isolated from the stem barks and fruits of *C. lansium* (Adebajo et al., 2009; Du et al., 2015; Fu et al., 2018; Liu et al., 2019). Notably, these alkaloids and coumarins exhibited impressive effects of anticancer and neuroprotection (Huang et al., 2016; Huang, Feng, Wang, & Lin, 2017; Iqbal et al., 2017; Ittiyavirah & Hameed, 2014; Liu et al., 2019). The seeds of wampee were also employed as folk medicine for the treatment of acute and chronic gastro‐intestinal disorders (Shen et al., 2018). Amide alkaloids isolated from the seeds of *C. lansium* showed potent nematicidal activity against *Panagrellus redivivus* (Fan et al., 2018) and antifungal effect against *Sclerotinia sclerotiorum* (Yan et al., 2018). Comparative assessment of phytochemical profiles and antioxidant activities in fruits of five varieties of wampee was conducted to demonstrate the difference of total phenolics, flavonoids, and antioxidant activities among different cultivars (Chang, Lu, et al., 2018; Chang, Ye, et al., 2018). Therefore, different parts of *C. lansium* include diverse bioactive ingredients and exhibit various bio‐functional values. However, to the best of our knowledge, the systematical analysis of the metabolites in different parts of *C. lansium* is limited that only the volatile components in leaf, pericarp, and seed of *C. lansium* have been characterized by GC‐MS (He et al., 2018). The lack of comprehensive analysis of phytochemical constitute of wampee might hinder its utilization as food supplements or potential pharmaceuticals in modern medicine.

Nowadays, metabolomics has become an important technology for the phytochemical profiling of biological metabolites with the development of liquid chromatography coupled to mass spectrometry. Until now, over 100,000 metabolites from plants have been detected which might be less than 10% of the total (Trethewey, 2004). Even though there is no single analytical method which can extract and identify all metabolites at one time, untargeted metabolomics aims to gather as many metabolites as possible (De Vos et al., 2007). The metabolites of various economical and/or medicinal plants such as lettuce (Garcia, García‐Villalba, Garrido, Gil, & Tomás‐Barberán, 2016), grape (Fig ueiredo et al., 2008), medicinal ginger (Jiang et al., 2006), *Acer truncatum* (Gu et al., 2019), and *Nigella* (Farag, Gad, Heiss, & Wessjohann, 2014) have been successfully characterized and quantified by untargeted metabolomics. With the rapid innovation in inter‐disciplinary technologies such as metabolomics, genomics, analytical chemistry, and computing, metabolic profiling is playing an important role in the areas of plants metabolic engineering (Trethewey, 2004), gene‐function analysis (Fernie, Trethewey, Krotzky, & Willmitzer, 2004), systems biology (Fernie et al., 2004), disease diagnosis (Fan et al., 2012), holistic quality evaluations of food products (Jandric et al., 2014), and traditional Chinese medicine (TCM) (Zhang et al., 2012).

The objective of the present study was to comprehensively analyze the metabolomics of the leaves (CLL), barks (CLBa), flowers (CLF), peels (CLPe), pulps (CLPu), and seeds (CLS) of *C. lansium* through applying untargeted metabolomics approach using liquid chromatography tandem mass spectrometry (UPLC‐Q‐Orbitrap‐MS). In order to compare the characteristic metabolites and identify the potential biomarkers, multivariate statistical analysis using bioinformatics tools was performed. A primary metabolic pathway analysis was also conducted to obtain putatively different metabolic biosynthesis pathway ascribing for the chemical composition of these six parts in wampee.

## 2 MATERIALS AND METHODS

### 2.1 Plant materials

The fruits, leaves, stems, and flowers of *C. lansium* were collected from the Wampee Resources Nursery of Institute of Fruit Tree Research, Guangdong Academy of Agricultural Sciences in Guangzhou, China. The pulps, peels, and seeds of the fruits were separated by a knife with caution and frozen in liquid nitrogen immediately. The barks were obtained from tender stems of *C. lansium*. All the plant materials were firstly pre‐treated with liquid nitrogen and then transferred to −80°C until extraction.

### 2.2 Chemicals

HPLC‐grade acetonitrile and methanol were purchased from Thermo Fisher Scientific Inc., and 2‐chlorobenzalanine was obtained from Aladdin Reagent Co., Ltd. All other chemicals were analytical grade and used as received.

### 2.3 Extraction

The extraction was conducted according to the method previously reported with minor modifications. Briefly, 200 mg samples were transferred into 5 ml tubes with five steel balls, and then they were placed into liquid nitrogen for 5 min before grind with a high flux organization grinding apparatus at 70 Hz for 1 min. Subsequently, 600 μl methanol (pre‐cooled at −20°C) was added and the mixture was vibrated for 30 s. And then, the extraction was carried out by an ultrasonicator for 30 min at room temperature. Afterward, 750 μl chloroform (pre‐cooled at −20°C) and 800 μl deionized water (4°C) was added into the tubes and shook for 60 s. After centrifugation at 13,523 *g* under 4°C for 10 min, the supernatant was obtained and lyophilized. The freeze‐dried samples were dissolved by 250 μl of a mixture containing 4 mg/ml 2‐chlorobenzalanine in methanol aqueous solution (1:1, 4℃). The dissolved samples were filtrated before detection by LC‐MS. For the quality control (QC) samples, 20 µl of each prepare sample extract was mixed all together.

### 2.4 LC‐MS conditions

The chromatographic separation was accomplished in a Thermo Ultimate 3,000 system equipped with an ACQUITY UPLC HSS T3 (150 × 2.1 mm, 1.8 µm, Waters) column which maintained at 40°C. The temperature of the autosampler was set at 8°C. Gradient elution of analytes was carried out with 0.1% formic acid in water (C) and 0.1% formic acid in acetonitrile (D) or 5 mmol/L ammonium formate in water (A), and acetonitrile (B) at a flow rate of 0.25 ml/min. Injection of 2 μl of each sample was done after equilibration. An increasing linear gradient of solvent B (v/v) was applied as follows: 0–1 min, 2%B/D; 1–9 min, 2%–50% B/D; 9–12 min, 50%–98% B/D; 12–13.5 min, 98% B/D; 13.5–14 min, 98%–2% B/D; 14–20 min, and 2% D‐positive model (14–17 min, 2% B‐negative model).

The electrospray ionization mass spectrometry experiments were executed on the Thermo Q Exactive Focus mass spectrometer (ThermoFisher) with the spray voltage of 3.8 kV and −2.5 kV in positive and negative modes, respectively. Sheath gas and auxiliary gas were set at 45 and 15 arbitrary units, respectively. The capillary temperature was 325℃, respectively. The Orbitrap analyzer scanned over a mass range of 81–1000 *m/z* for full scan at a mass resolution of 70,000. Data‐dependent acquisition (DDA) MS/MS experiments were performed with HCD scan. The normalized collision energy was 30 eV. Dynamic exclusion was implemented to remove some unnecessary information in MS/MS spectra. Six biological replicates were conducted for each sample.

### 2.5 Mass data processing and multivariate data analyses

Raw LC‐MS data were converted into mzXML format files via Proteowizard Data Analysis software (v3.0.8789) and subsequently processed peaks identification, peaks filtration, and peaks alignment via XCMS (www.bioconductor.org). XCMS’s default set with the following changes: bw = 2, ppm = 15, peakwidth = c(5, 30), mzwid = 0.015, mzdiff = 0.01, and method = centWave. Each metabolite was confirmed based on their exact molecular weights, and the possible empirical formulae of the metabolites were speculated (molecular weight error <20 ppm). The exact molecular weights were then used to identify potential biomarkers by confirmation in the Human Metabolome Database (HMDB) (http://www.hmdb.ca), Metlin (http://metlin.scripps.edu), massbank (http://www.massbank.jp/), Lipid Maps (http://www.lipidmaps.org), mzclound (https://www.mzcloud.org), and database built by Bionovogene Co., Ltd.

For multivariate statistical analysis, the XCMS output was further processed using Microsoft Excel (Microsoft), and the normalized data were imported into the Simca‐P software version 11.0 (Umetrics AB, www.umetrics.com/simca). All data were mean‐centered and unit variance (UV)‐scaled before PCA and PLS–DA applied in order to guard against overfitting. A default 7‐fold (Leave‐1/7th samples‐Out) cross‐validation procedure and 100 random permutations testing were carried out to guard against overfitting of supervised PLS‐DA models. These discriminating metabolites were obtained using a statistically significant threshold of variable influence on projection (VIP > 1.0). Values were obtained from the PLS–DA model and were further validated via Student's *t* test (*p* < .05). The metabolites with VIP values above 1.0 and *p* values below .05 (threshold) were selected as discriminating metabolites between two classes of samples. Multivariate data analyses including principal components analysis (PCA), partial least squares discriminant analysis (PLS‐DA), and Orthogonal PLS‐DA (OPLS‐DA) were conducted using the *ropls* R (version 3.3.2) package with methods described previously.(Thévenot, Roux, Xu, Ezan, & Junot, 2015) The *ropls* package is available from the Bioconductor repository (Gentleman et al., 2004). Discriminating metabolites between 2 classes of samples were identified using a statistically significant threshold of Variable Importance in Projection (VIP) value (VIP ≥ 1), and further validated by one‐way univariate analysis of variance (ANOVA) value (*p* ≤ .05).

### 2.6 Heat map and KEGG annotation

Heat map was constructed using Euclidian distances and complete linkage grouping with the pheatmap package in R language (www.r-project.org), and the relative quantitative values of metabolites were normalized, transformed, and clustered through agglomerate hierarchical clustering. Metabolite correlation was assessed using Pearson Correlation Coefficient and constructed Cytoscape software (www.cytoscape.org). To further identify alternative metabolic pathways, differential metabolites were subjected to grouping and enrichment of metabolic pathway using MetaboAnalyst 4.0 software (www.metaboanalyst.ca) and KEGG database (www.kegg.jp). The identified differential metabolites were reacted to biochemical pathways according to the labeling in KEGG (http://www.kegg.jp/pathway). Metabolic pathway enrichment and topological analysis were performed using the MetPA database (www.metaboanalyst.ca) to analyze metabolic pathways related to two different metabolites.

## 3 RESULTS AND DISCUSSION

### 3.1 Metabolites identification

In order to ensure the validation of results obtained from untargeted large‐scale metabolomics, quality control (QC) and quality assurance (QA) were performed for the first place. The results of QC were illustrated in Figure S1 A,B which indicated that the extraction and detection of samples were stable.(Dunn et al., 2011) The ratio of characteristic peaks which the relative standard deviation (RSD) is <30% can reach about 70% (Figure S1 C,D), indicating that the data are reliable.(Want et al., 2010) After obtained, the information of m/z (mass to charge ratio), rt (retention time) and intensity, 15,635 and 18,064 precursor molecules were detected in positive mode and negative mode, respectively. Batch normalization was employed for all the data. In the present study, a total of 364 metabolites were identified from all the samples of wampee and the detailed information including their retention time, exact mass, molecular formula, Precursor type, match percentage, CAS number, and KEGG code was provided in Table S1. As shown in Table S1, there are 100 organic acids, 47 amino acids and derivates, 45 flavonoids, 28 nucleotides and derivates, 24 lipids, 19 alcohols, 17 amines, 15 carbohydrates, 13 vitamins and derivates, 11 alkaloids, 10 peptides, 5 coumarins, 4 aldehydes, 3 ketones, 2 indole derivatives, 1 terpene, and 20 other metabolites identified from different parts of *C. lansium*. Organic acids are the main constituents in *C. lansium*, followed with amino acids and flavonoids. Flavonoids were proved to be powerful radical quenchers in various systems.(Zhang et al., 2018) Most of the flavonoids were firstly identified in *C. lansium* such as Procyanidin B2, malvidin 3‐glucoside, isoquercitrin, astragalin, quercetin 3‐arabinoside, taxifolin, sakuranetin, etc.. Procyanidin B2, and malvidin 3‐glucoside mainly derived from grape seed and red wine were proved to exert protective effects against cardiovascular diseases (Bub, Watzl, Heeb, Rechkemmer, & Briviba, 2001; Li & Zhu, 2019). Isoquercitrin (quercetin‐3‐O‐β‐d‐glucopyranoside) and astragalin commonly found in traditional herbs and medicinal plants were reported to have anti‐inflammatory effects (Rogerio et al., 2007; Soromou et al., 2012; Valentova, Vrba, Bancirova, Ulrichova, & Kren, 2014). Quercetin 3‐arabinoside and delphinidin‐3‐O‐arabinoside were also identified in Chilean *Gaultheria* berry (Mieres‐Castro et al., 2019) and bilberry (*Vaccinium myrtillus* L.) (Liu, Laaksonen, Yang, Zhang, & Yang, 2020). Sinomenine, identified in this study, is a bioactive alkaloid which has been used as a treatment of rheumatoid diseases. (Zhang, Zhang, Zheng, & Tian, 2019) Vindoline, an indole alkaloid with anticancer activity, which is derived from *Catharanthus roseus,* was also detected. (Taher et al., 2019) Therefore, the global identification of metabolites in different parts of wampee may provide new insights for the understanding of the bioactivities of *C. lansium*.

### 3.2 Biomarker probe

**FIGURE 1:**
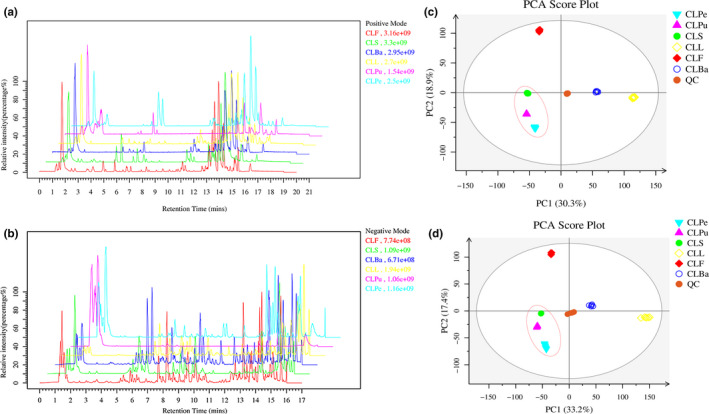
Base peak ion (BPI) chromatograms of different parts of wampee in positive (a) and negative (b) modes. Principal component analysis (PCA) of different parts of wampee in positive (c) and negative (d) modes

As illustrated in the base peak ion (BPI) chromatograms of different parts of wampee (Figure 1a,b), the differences between samples were evident as observed in both negative and positive ion modes. In the current study, multivariate statistical analysis was carried out by unsupervised principal component analysis (PCA) to firstly observe the overall distribution between samples and original state of the data under reduced dimensionality. (Lever, Krzywinski, & Altman, 2017) As shown in Figure 1c,d of the PCA score plots in positive and negative ion modes, the QC samples were clustered closely and were near the center of the plots which suggested that the data are in good state. In addition, the variation among different parts of wampee can be confirmed not generated by system deviations. Notably, CLS, CLPu, and CLPe which composed the whole fruits were clustered closely (Figure 1c,d) indicating that different parts of wampee fruits might contain similar chemical components especially the pulp and the peel. CLF is far from other parts suggesting that the metabolic profile of wampee flower is unique. According to the results of PCA, we can infer that the metabolites of different parts of wampee were distinct from each other to some degree.

**FIGURE 2:**
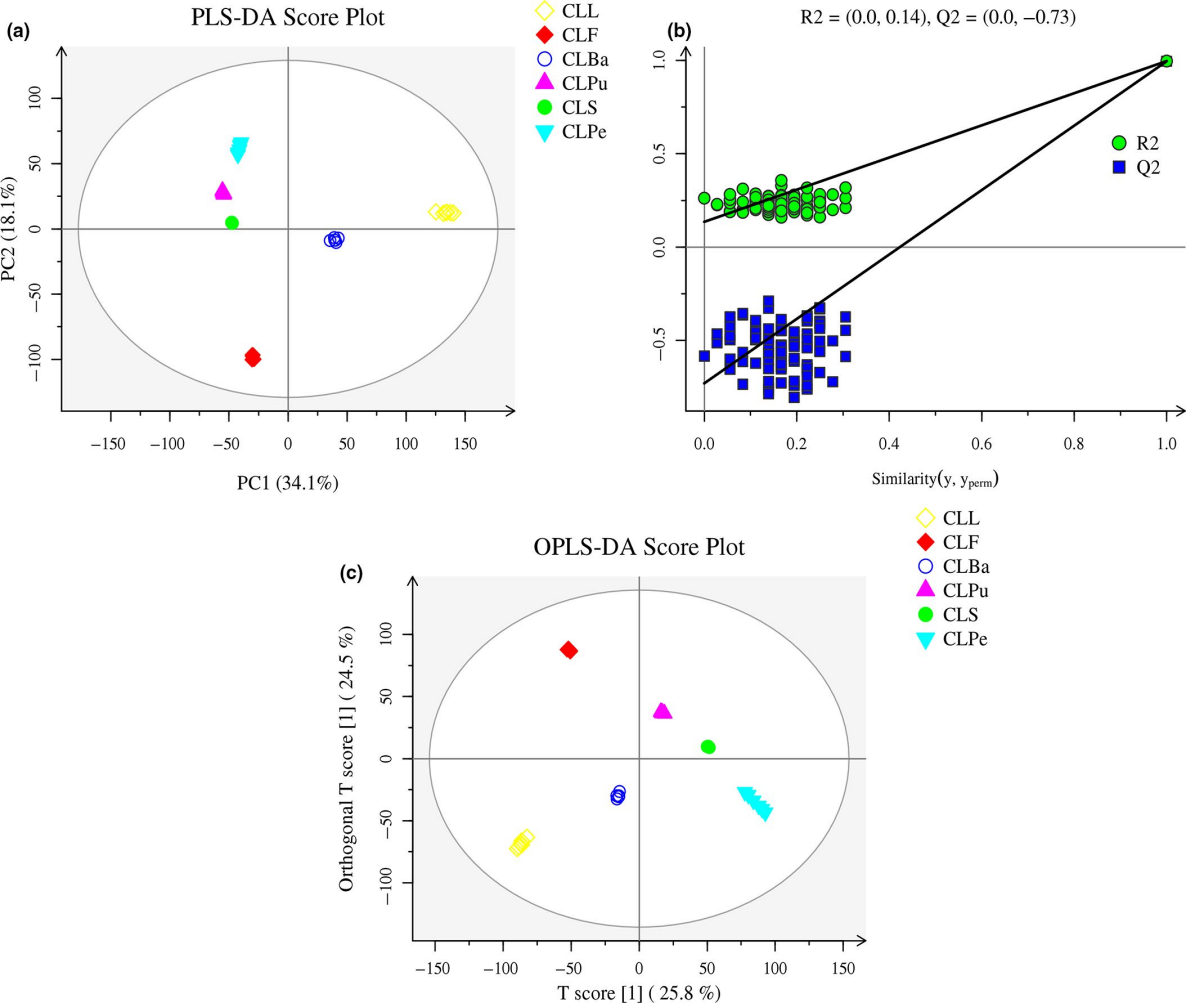
Partial least squares‐discriminate analysis (PLS‐DA) of different parts of wampee in negative mode (a). Permutations plot of the PLS‐DA model for the CLL versus CLF versus CLBa versus CLPu versus CLS versus CLPe (b). Orthogonal projections to latent structures discriminant analysis (OPLS‐DA) of different parts of wampee in negative mode (c). (CLF, CLBa, CLL, CLPe, CLPu, and CLS represent the flowers, barks, leaves, peels, pulps, and seeds of wampee)

However, unsupervised analysis, such as PCA, cannot ignore intra‐group errors, eliminate random errors that are irrelevant to the purpose of the study, and it pays too much attention to details which ignore the whole rules of the data, and ultimately, it cannot distinguish find differences and differential compounds between groups. In this case, it is necessary to apply supervised analysis, such as partial least squares‐discrimination analysis (PLS‐DA) and orthogonal projections to latent structures discriminant analysis (OPLS‐DA) for the further probe of biomarkers among different parts of wampee. In Figure 2a, plot of PLS‐DA indicates the significant differences among different groups and good repetition of the data in individual groups. Nevertheless, overfitting is usually existed during the modeling of PLS‐DA. The permutations test was employed for the evaluation of statistical significance of the model. As shown in Figure 2b, R2, and Q2 are similar, indicating that each of the subjects contributes equally and uniformly to the observed group separation (Wheelock & Wheelock, 2013). Hence, the modeling of PLS‐DA utilized in this study is stable and reproducible.

OPLS‐DA and S‐plot were applied to different compared groups to identify biomarkers among different parts of wampee. The scatter score plot of OPLS‐DA inferred from the comparison of all the samples in was presented in Figure 2c, and the detailed parameters of OPLS‐DA for different compared groups were shown in Table 1. As can be seen from Figure 2c and Table 1, differences among CLL, CLF, CLBa, CLPu, CLS, and CLPe were significant and each model has high R2 and Q2 values suggesting that the models are reliable. Therefore, the results of multivariate statistical analysis demonstrate significant differences in metabolites among different parts of wampee. The identification of significantly changing potential biomarkers was filtered by means of ANOVA *p* value ≤.05 and VIP ≥ 1. The information of the differential metabolites compared in various groups was offered in Table S2. In addition, S‐plots were conducted for all the compared models (shown in Figure S2) to further probe the significant marker compounds. In Figure S2, the variables with significant differences will be plotted at the top right and the bottom left, and the ones with no significant differences will appeared in the middle of the plot. Under the help of the multivariate statistical analysis, as a result, a total of 62 potential biomarkers of different parts of wampee were selected and summarized in Table 2. The selected differential metabolites include 16 organic acid, 10 flavonoids, 9 amino acids, 6 lipids, 4 alkaloids, 4 nucleotides, 2 carbohydrates, 2 vitamins, 2 peptides, 2 alcohols, 1 terpene, 1 coumarin, and 3 others.

**TABLE 1:** Values of the statistic parameters obtained for different OPLS‐DA models based on LC‐MS data (in negative mode)^a^

Model classes	OPLS‐DA				Number of DM^a^
	pre	R2X cum, %)	R2Y cum, %)	Q2 cum, %)	
CLF versus CLL	1+1+0	76.5	100.0	99.8	243
CLF versus CLBa	1+1+0	71.8	100.0	99.7	244
CLBa versus CLL	1+1+0	68.9	100.0	99.5	215
CLPu versus CLPe	1+1+0	68.6	100.0	98.8	212
CLPu versus CLS	1+1+0	63.9	100.0	99.4	230
CLS versus CLPe	1+1+0	70.2	100.0	99.3	235
CLPe versus CLPu versus CLS	1+1+0	72.2	99.6	99.1	194
CLPu versus CLPe versus CLS versus CLF	1+1+0	50.9	99.9	99.7	198
CLPu versus CLPe versus CLS versus CLBa	1+1+0	57.4	99.4	99.0	161
CLPu versus CLPe versus CLS versus CLL	1+2+0	83.3	99.9	99.6	175
CLL versus CLF versus CLBa versus CLPu versus CLS versus CLPe	1+2+0	58.9	99.8	99.6	140

^a^ Pre is the number of principal components; R2X is the model interpretability (for X variable dataset); R2Y is the model interpretability (for Y variable dataset); Q2 is the percentage of model predictability, DM means differential metabolites selected by VIP > 1, and the detailed information of the DM was provided in Table S2.

**TABLE 2:** Potential biomarkers of different parts of wampee

No.	Name	RT	Formula	CLL	CLF	CLBa	CLPu	CLS	CLPe
1	β‐Alanine	91.36	C_3_H_7_NO_2_				√	√	√
2	4‐Pyridoxic acid	240.83	C_8_H_9_NO_4_	√	√	√		√	
3	Phosphorylcholine	734.48	C_5_H_15_NO_4_P		√	√	√		√
4	N‐Acetylglutamic acid	96.50	C_7_H_11_NO_5_	√	√	√	√		
5	N‐Methyltyrosine	233.87	C_10_H_13_NO_3_	√	√	√		√	
6	(‐)‐α‐Curcumene	446.50	C_15_H_22_	√		√		√	√
7	Acetylcarnitine	152.16	C_9_H_17_NO_4_	√	√	√		√	
8	Propionylcarnitine	306.06	C_10_H_19_NO_4_	√		√			
9	Hydroxykynurenine	705.72	C_10_H_12_N_2_O_4_		√	√	√		√
10	Butyryl‐L‐carnitine	389.53	C_11_H_21_NO_4_	√	√	√			√
11	Nicotinamide riboside	107.58	C_11_H_15_N_2_O_5_	√	√	√			√
12	Acoric acid	714.32	C_15_H_24_O_4_	√	√	√			√
13	Naringenin	695.10	C_15_H_12_O_5_	√	√	√		√	
14	13‐epi‐12‐oxo Phytodienoic acid	752.66	C_18_H_28_O_3_	√	√	√		√	
15	(R)‐Oxypeucedanin	579.81	C_16_H_14_O_5_	√		√		√	√
16	8,11,14‐Eicosatrienoic acid	547.13	C_20_H_34_O_2_	√		√			√
17	Glutathione	110.44	C_10_H_17_N_3_O_6_S			√			√
18	2,3‐dinor Prostaglandin E1	738.23	C_18_H_30_O_5_	√	√	√			
19	γ‐Glutamyltyrosine	342.27	C_14_H_18_N_2_O_6_	√	√	√			
20	13(S)‐HpOTrE	688.31	C_18_H_30_O_4_	√	√	√		√	
21	Sinomenine	674.22	C_19_H_23_NO_4_	√	√				
22	3‐AMP	150.27	C_10_H_14_N_5_O_7_P		√		√	√	√
23	Allocholic acid	456.61	C_24_H_40_O_5_	√	√	√		√	
24	Delphinidin‐3‐O‐arabinoside	537.02	C_20_H_19_O_11_	√	√	√			√
25	Astragalin	524.21	C_21_H_20_O_11_	√	√			√	
26	Quercitrin	466.07	C_21_H_20_O_11_		√	√			√
27	Trp‐Lys‐OH	532.42	C_23_H_26_N_4_O_6_	√	√	√		√	
28	Hesperetin 7‐O‐glucoside	462.71	C_22_H_24_O_11_	√	√	√			
29	Malvidin 3‐glucoside	558.74	C_23_H_25_O_12_				√	√	√
30	Leukotriene F4	599.90	C_28_H_44_N_2_O_8_S	√	√	√			√
31	Benzene‐1,2,4‐triol	535.46	C_6_H_6_O_3_		√	√		√	
32	Ketoleucine	342.94	C_6_H_10_O_3_	√	√	√		√	
33	Hypoxanthine	201.02	C_5_H_4_N_4_O		√	√	√	√	
34	4‐Hydroxybenzoic acid	164.19	C_7_H_6_O_3_		√	√	√		√
35	Oxoglutaric acid	165.12	C_5_H_6_O_5_	√	√	√		√	
36	D‐beta‐Phenylalanine	638.67	C_9_H_11_NO_2_	√	√	√			√
37	Gallic acid	318.50	C_7_H_6_O_5_				√		√
38	4‐hydroxynonenoic acid	818.81	C_9_H_16_O_3_	√	√	√		√	
39	N‐Acetylleucine	159.55	C_8_H_15_NO_3_			√	√	√	√
40	N‐α‐acetyllysine	222.95	C_8_H_16_N_2_O_3_	√	√	√			
41	10‐Hydroxydecanoic acid	584.56	C_10_H_20_O_3_		√	√		√	√
42	Pantothenic acid	227.10	C_9_H_17_NO_5_		√	√	√		√
43	Dodecanedioic acid	476.69	C_12_H_22_O_4_	√	√			√	√
44	Alantolactone	707.74	C_15_H_20_O_2_	√	√	√			
45	5,7‐Dihydroxyflavone	778.00	C_15_H_10_O_4_					√	√
46	Medicarpin	794.97	C_16_H_14_O_4_	√	√	√			√
47	Asp‐Phe	274.87	C_13_H_16_N_2_O_5_	√	√	√			
48	Biochanin A	775.04	C_16_H_12_O_5_		√	√		√	√
49	Melibiose	232.99	C_12_H_22_O_11_	√		√	√	√	
50	Nicotinamide ribotide	689.10	C_11_H_15_N_2_O_8_P		√	√		√	
51	Aesculin	400.14	C_15_H_16_O_9_	√	√	√			√
52	Dehydro‐L‐(+)‐ascorbic acid dimer	792.02	C_20_H_19_NO_5_		√	√	√	√	
53	Parfumine	326.34	C_15_H_16_O_11_	√		√			
54	Secoisolariciresinol	590.04	C_20_H_26_O_6_	√	√	√			√
55	Linustatin	406.43	C_19_H_28_O_11_	√		√	√		√
56	Deoxycholic acid	815.64	C_24_H_40_O_4_		√	√	√		√
57	Quercetin 3‐arabinoside	531.68	C_20_H_18_O_11_	√	√	√			√
58	Raffinose	514.83	C_18_H_32_O_16_	√		√			
59	dTDP‐3‐O‐methyl‐beta‐L‐rhamnose	399.53	C_17_H_28_N_2_O_15_P_2_	√	√	√			√
60	NAD	218.69	C_21_H_28_N_7_O_14_P_2_		√		√	√	√
61	Quercetin 3‐(2G‐xylosylrutinoside)	450.79	C_32_H_38_O_20_	√	√				√
62	p‐Coumaroyl‐CoA	413.78	C_30_H_42_N_7_O_18_P_3_S		√	√	√		√

### 3.3 Hierarchical cluster analysis

**FIGURE 3:**
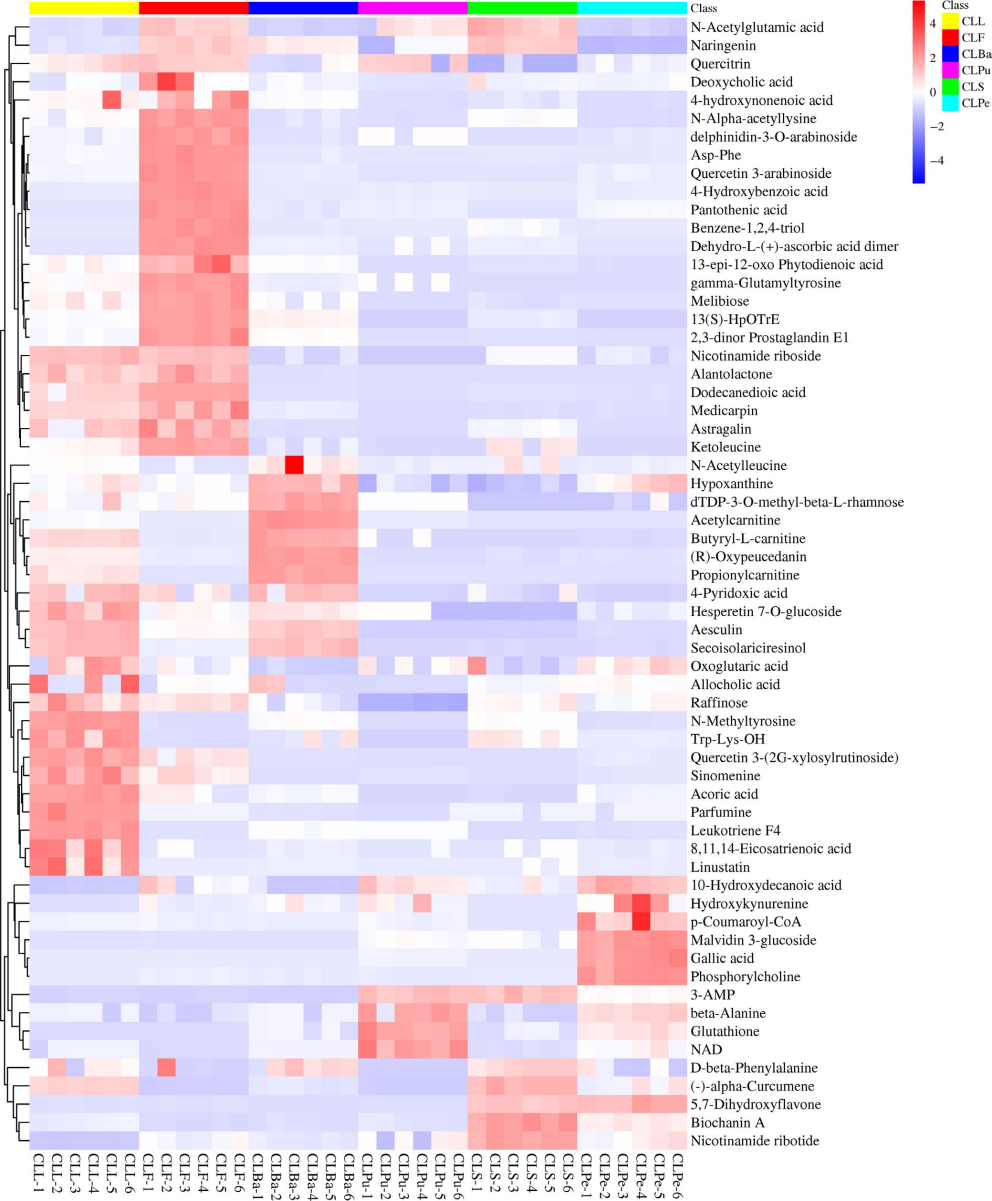
Heatmap of hierarchical clustering analysis of differential metabolites selected as the biomarkers in different parts of wampee. The abscissa indicates different groups labeled with different color for the main groups and numerically marked for the subgroups. The ordinate indicates the differential metabolites selected as the potential biomarkers in different parts of wampee. The bar at the right of the heat map represents relative expression values

In order to visualize the relative contents of the differential metabolites in different parts of wampee and classify the metabolites with similar characteristics, hierarchical cluster analysis (HCA) of 62 potential biomarkers was conducted and the result was illustrated with heat map as shown in Figure 3. The color squares changed from red to blue indicate the decreasing amount of the metabolites. The HCA was conducted for all the samples including the 6 biological duplications, and the color of them is relatively uniform indicating good repeatability and reliable data. Moreover, the HCA of differential metabolites of different parts of wampee demonstrated a clear grouping pattern. The contents of some flavonoids such as quercitrin, delphinidin‐3‐O‐arabinoside, quercetin 3‐arabinoside, and astragalin were upregulated in the flower of *C. lansium*. Aesculin and hesperetin 7‐O‐glucoside which are the precursor of neohesperedin/hesperidin have a relatively large amount in the leaves compared with other parts of *C. lansium*. The content of malvidin 3‐glucoside and gallic acid in the peel of wampee is the highest. The pulp of wampee contains the highest level of glutathione and β‐alanine, and the seed contains relatively abundant naringenin and biochanin A. 5,7‐Dihydroxyflavone is abundant in both the peel and the seed of wampee fruits. The result of hierarchical cluster analysis suggested that the selected biomarkers could be utilized for the differentiation different parts of wampee.

### 3.4 KEGG annotation and metabolic pathway analysis

The metabolites in plant are usually synthesized through complex metabolic reactions under the help of different genes and proteins which form complex pathways and networks. And the secondary plant metabolites are determined by both the genus and the environment. In the present study, all the samples were collected from the same orchard at the same time; hence, different metabolites detected were produced by the regulation of the genes in different tissues of wampee. KEGG (Kyoto Encyclopedia of Genes and Genomes) is a database that integrates genomic, chemical, and system functional information, one of the database of KEGG is LIGAND, which contains information about chemicals, enzyme molecules, and enzyme reactions (Kanehisa & Goto, 2000). The KEGG database was employed to map and define the metabolic pathways of the differential metabolites in various comparison groups using *A. thaliana* as the library.

**FIGURE 4:**
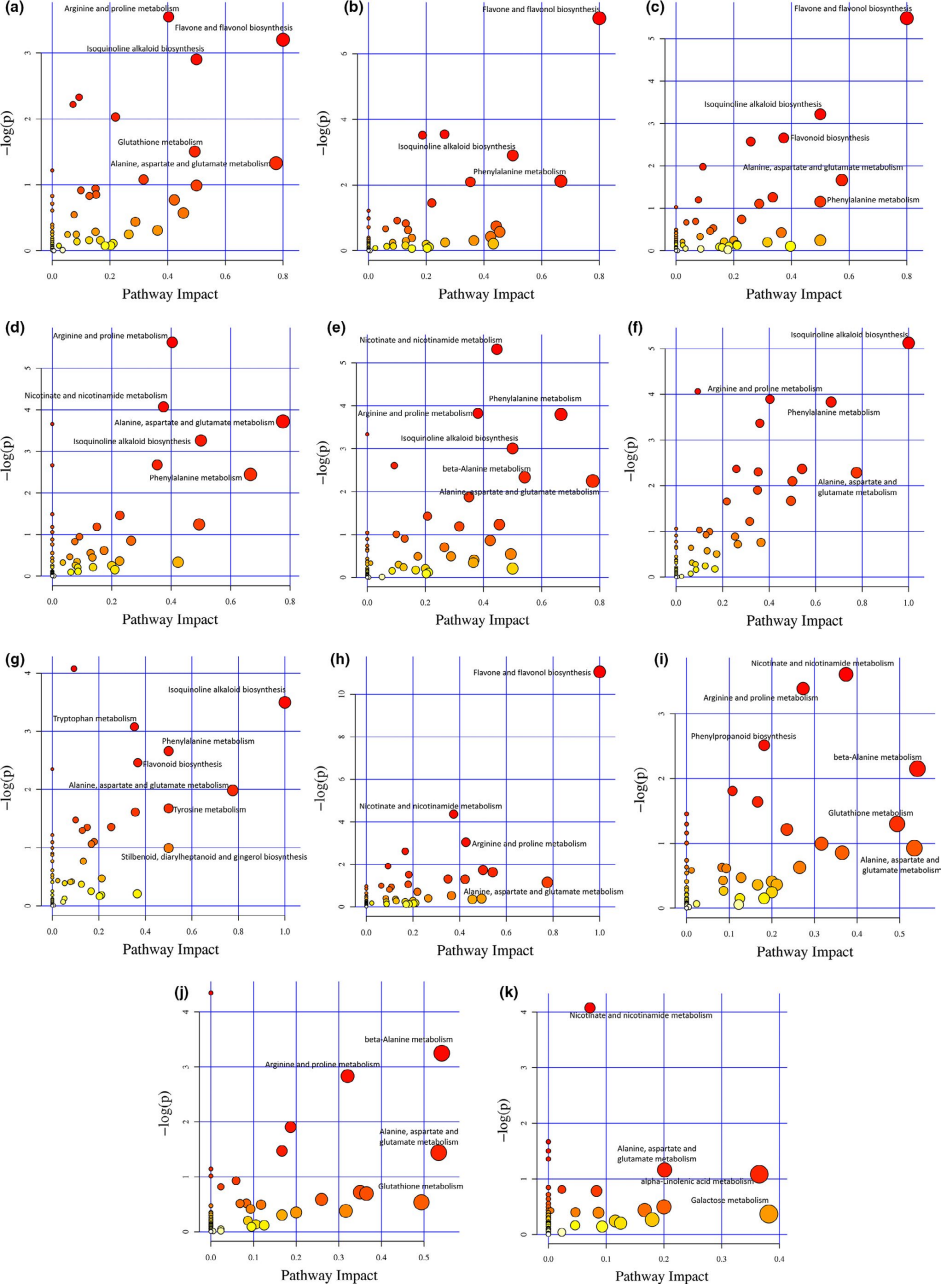
Pathway analysis of differential metabolites for CLF versus CLL (a), CLF versus CLBa (b), CLBa versus CLL (c), CLPu versus CLPe (d), CLPu versus CLS (e), CLS versus CLPe (f), CLPu versus CLPe versus CLS (g), CLPe versus CLPu versus CLS versus CLF (h), CLPe versus CLPu versus CLS versus CLBa (i), CLPe versus CLPu versus CLS versus CLL (j), CLL versus CLF versus CLBa versus CLPe versus CLPu versus CLS (k). (CLF, CLBa, CLL, CLPe, CLPu, and CLS represent the flowers, barks, leaves, peels, pulps, and seeds of wampee) Each bubble in the plot represents a metabolic pathway whose abscissa and bubble size jointly indicate the magnitude of the impact factors of the pathway in the topological analysis. The bubble ordinates and colors represent the *p* values (negative natural logarithm, i.e., −log *p*‐value) of the enrichment analysis, with darker colors showing a higher degree of enrichment. The most significant pathway was labeled

In this study, pathway analysis was carried out for all the 11 compared groups, and the detailed results were provided in Table S3. And a bubble plot with the most significant pathway marked was also presented in Figure 4. The impact factor in Figure 4 was defined as the number of metabolites mapped to a certain pathway versus to the total number of metabolites mapped to this pathway. The significance of the pathway in compared groups was judged by both the impact factor and the *p* value, in other words is the pathway located in the most upper right corner is the most significant. Flavone and flavonol synthesis was considered as the main enrichment metabolic pathway for the differential metabolites in the 4 compared groups of CLF versus CLL, CLF versus CLBa, CLBa versus CLL, and CLPe versus CLPu versus CLS versus CLF with *p* value <.05 and impact factor > 0.8. The most significant metabolic pathway in groups of CLS versus CLPe, and CLPu versus CLPe versus CLS is the isoquinoline alkaloid biosynthesis (*p* < .05, impact factor = 1). As shown in Figure 4, isoquinoline alkaloid biosynthesis is also an important metabolic pathway in groups of CLBa versus CLL, CLPu versus CLPe, CLPu versus CLS with *p* < .05 and impact factor = 0.5. In CLPu versus CLPe, CLPu versus CLS, CLPe versus CLPu versus CLS versus CLBa, CLPe versus CLPu versus CLS versus CLF, and CLL versus CLF versus CLBa versus CLPe versus CLPu versus CLS, the metabolite differences enriched metabolic pathway involves the nicotinate and nicotinamide metabolism (*p* < .05). Phenylalanine metabolism is a significant pathway in groups of CLF versus CLBa, CLBa versus CLL, CLPu versus CLPe, CLPu versus CLS, CLS versus CLPe and CLPu versus CLPe versus CLS with the impact factor ≥ 0.5. And similarly, another important pathway with the impact factor > 0.5 in almost all the compared groups except CLF versus CLBa and CLL versus CLF versus CLBa versus CLPe versus CLPu versus CLS is the alanine, aspartate, and glutamate metabolism. In consequence, the “Flavone and flavonol synthesis,” “Isoquinoline alkaloid biosynthesis,” “Nicotinate and nicotinamide metabolism,” “Phenylalanine metabolism,” and “Alanine, aspartate and glutamate metabolism” have been identified as important metabolic pathways for the formation of tissue difference of metabolites in *C. lansium*. Furthermore, the KEGG annotation and metabolic pathway analysis (Figure 4, Table S3) are consistent with the result that there are 10 flavonoids, 9 amino acids, 4 alkaloids, and 4 nucleotides differential metabolites different parts of wampee.

## 4 CONCLUSIONS

The comparative non‐targeted large‐scale plant metabolomics approach was carried out for the evaluation of the biomarkers of different tissues from *C. lansium*. To the best of our knowledge, this is the first report about the metabolism of wampee, and a total of 364 metabolites were identified. The results of PLS, OPLS‐DA, S‐plot, and HCA suggested that the selected 62 chemicals as potential biomarkers can be utilized for the differentiation of the leaves, barks, flowers, pulps, peels, and seeds of wampee. Metabolic pathways of “Flavone and flavonol synthesis,” “Isoquinoline alkaloid biosynthesis,” “Nicotinate and nicotinamide metabolism,” “Phenylalanine metabolism,,” and “alanine, aspartate, and glutamate metabolism” are important for the synthesis of differential metabolites among the different tissues of *C. lansium*. Therefore, on one hand, this investigation could provide good basis for the isolation and identification of new constituents from *C. lansium*; on the other hand, further detailed elucidation of the biosynthesis of certain differential metabolites among various tissues of wampee could be conducted accordingly in the future.

## Supporting information

Fig S1‐S2

Table S1

Table S2

Table S3
