# Supplementary material for: A comparative UPLC‐Q‐Orbitrap‐MS untargeted metabolomics investigation of different parts of Clausena lansium (Lour.) Skeels
Source: Food Sci Nutr. 2020 Oct 3;8(11):5811–22. doi: 10.1002/fsn3.1841 (PMC7684609; doi:10.1002/fsn3.1841)
Supplement: Supplementary file 1 — Fig S1‐S2 [file FSN3-8-5811-s001.docx]

**Supplementary Materials**

**A comparative UPLC-Q-Orbitrap-MS untargeted metabolomics investigation of different parts of** ***Clausena lansium* (Lour.) Skeels**

Ruiyi Fan, Cheng Peng, Xinxin Zhang, Diyang Qiu, Genlin Mao, Yusheng Lu*, Jiwu Zeng*

Institute of Fruit Tree Research, Guangdong Academy of Agricultural Sciences; Key Laboratory of South Subtropical Fruit Biology and Genetic Resource Utilization (MOA); Guangdong Province Key Laboratory of Tropical and Subtropical Fruit Tree Research, Guangzhou, 510640, China

*Corresponding authors: luyusheng6702746@126.com (Y. L.); jiwuzeng@163.com (J. Z.)


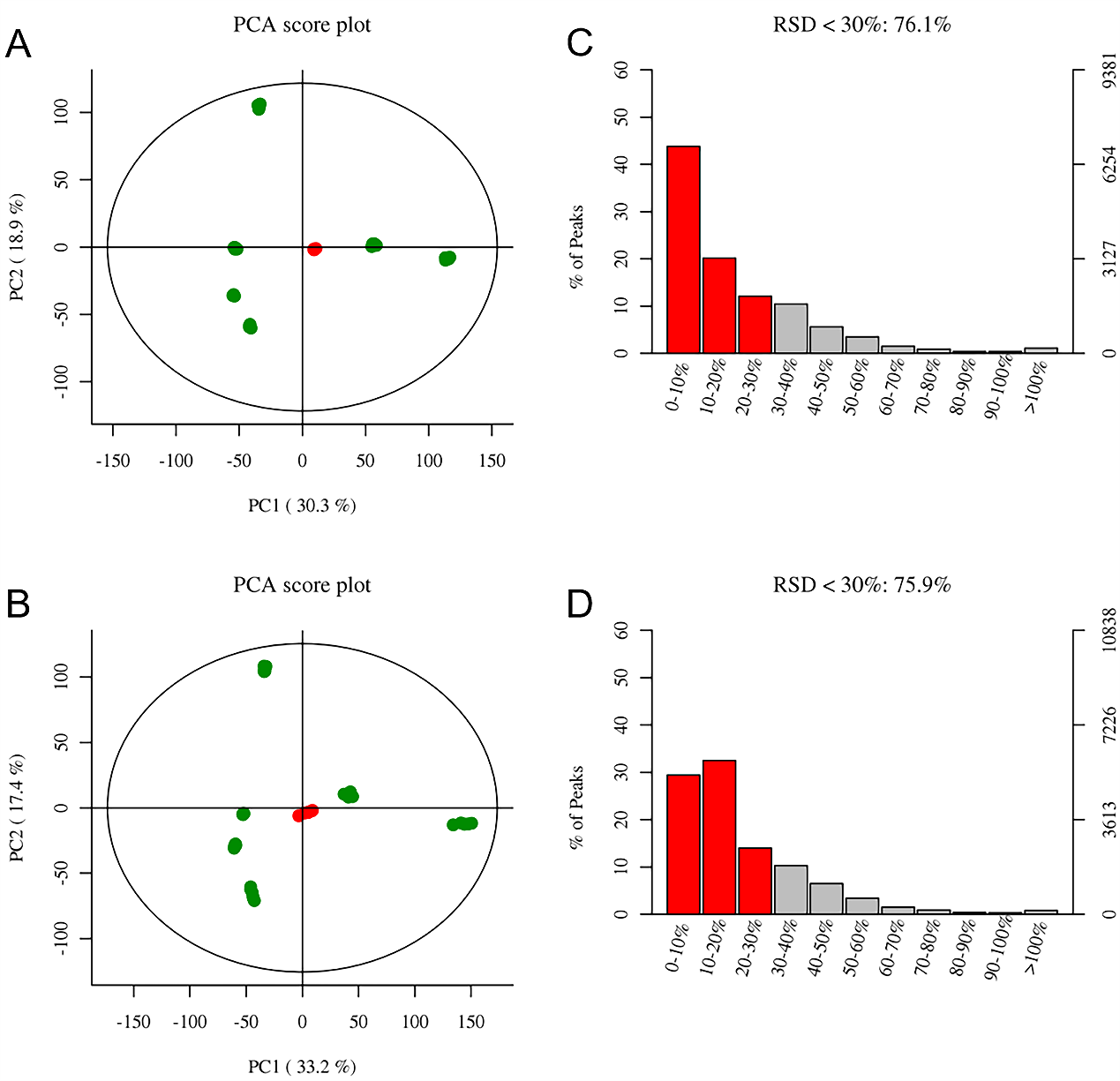


**Figure S1** Score scatter plots for principal component analysis (PCA) model with all the samples represent with green dots and the quality control (QC) represent with red dots in positive mode (A) and negative mode (B). Relative standard deviation (RSD) tests of the detected peaks in positive mode (C) and negative mode (D).


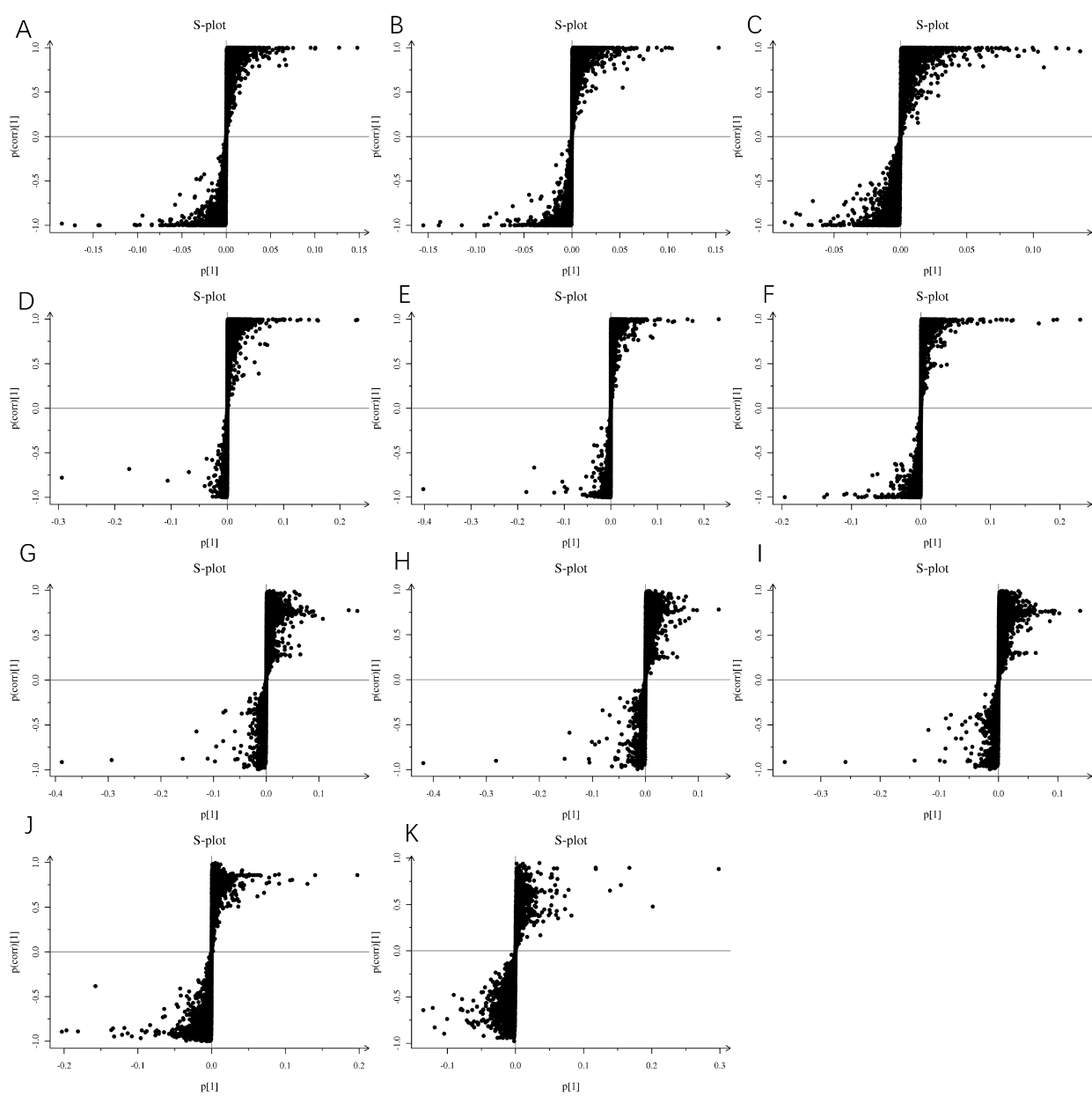


**Figure S2** S-plots of the comparison models of CLF vs CLL (A), CLF vs CLBa (B), CLBa vs CLL (C), CLPu vs CLPe (D), CLPu vs CLS (E), CLS vs CLPe (F), CLPu vs CLPe vs CLS (G), CLPe vs CLPu vs CLS vs CLF (H), CLPe vs CLPu vs CLS vs CLBa (I), CLPe vs CLPu vs CLS vs CLL (J), CLL vs CLF vs CLBa vs CLPe vs CLPu vs CLS (K).

**Supplementary Tables caption**

**Table S1.** All compounds identified from *Clausena lansium* (Lour.) Skeels

**Table S2.** The information of the differential metabolites compared in various groups (information of each group was provided in individual sheets)

**Table S3.** The KEGG annotation and metabolic pathway analysis compared in various groups (information of each group was provided in individual sheets)
